# CAFs and Endocrine Therapy Resistance in Hormone Receptor-Positive Breast Cancer

**DOI:** 10.3390/ijms27104633

**Published:** 2026-05-21

**Authors:** Amalia A. Sofianidi, Vaia K. Stafyla, Flora Zagouri

**Affiliations:** 1 Oncology Unit, Aretaieion University Hospital, School of Medicine, National and Kapodistrian University of Athens, 11527 Athens, Greece; florazagouri@yahoo.com.uk; 2 4th Department of Surgery, Attiko Hospital, School of Medicine, National and Kapodistrian University of Athens, 12462 Athens, Greece; vaniastafyla@hotmail.com

**Keywords:** cancer-associated fibroblasts, CAFs, endocrine resistance, luminal, breast cancer

## Abstract

The development of endocrine resistance represents a major obstacle when treating hormone receptor-positive breast cancer. The tumor microenvironment (TME), represented by cancer-associated fibroblasts (CAFs) in this context, has recently been proposed as a key mediator significantly contributing to resistance against currently available endocrine therapies. The exact mechanisms behind this interaction are not fully understood; specific breast CAF subtypes have been linked to it, such as CAFs lacking the expression of the glycoprotein CD146 or maintaining the expression of CD63. Other proposed mechanisms include signaling pathways aberrantly activated in CAFs, epigenetic modifications mainly in the form of long non-coding RNAs (lncRNAs) and microRNAs (miRNAs), and paracrine signaling, all limiting endocrine modulation effectiveness. Strategies aiming to simultaneously target CAFs and endocrine signaling in luminal breast cancer are currently being developed. Fibroblast growth factor receptor (FGFR) targeting in combination with endocrine inhibition has already entered the clinical trial landscape. However, CAFs are a highly diverse and heterogeneous cell population, making their targeting complex and difficult to implement in clinical practice.

## 1. Introduction

In 2022, 2.3 million new cases and 670,000 deaths from female breast cancer were reported globally. However, what is even worse is that by 2050, new breast cancer cases and deaths will have increased by 38% and 68%, respectively [1]. Approximately 70–80% of newly diagnosed breast cancer cases are hormone receptor-positive (HR+), characterized by the expression of the estrogen receptor (ER) with or without a concurrent expression of the progesterone receptor (PR) [2]. Endocrine therapy is the cornerstone of HR+ breast cancer, consisting of selective estrogen receptor modulators (SERMs), selective estrogen receptor degraders (SERDs) or aromatase inhibitors (AIs) in combination with targeted agents like Cyclin-Dependent Kinases 4 and 6 (CDK4/6) inhibitors when high-risk or advanced disease is present [3].

However, despite the available treatment options, which aim at depriving cancer cells from hormone signaling, the possibility of recurrence is substantial. A recently published meta-analysis determines the 3-year recurrence rate for patients with HR+, human epidermal growth factor receptor 2 negative (HER2−) breast cancer at 7%, regardless of lymph node status and overall stage [4]. Looking further, the 5- and 10-year risk of recurrence in patients with node-negative breast cancer is 22.1% and 36.9%, respectively [5]. In contrast, when lymph node infiltration is present, the risk increases to 28.9% and 49.4%, respectively [5]. Given that the incidence of breast cancer in younger ages is rising, the aforementioned rates make it clear that more effective treatment options are required [6].

The interest of the scientific community has recently shifted to the tumor microenvironment (TME), an ecosystem where malignant cells flourish. This dynamic ecosystem is rich in various components, consisting of immune cells, stromal cells, blood vessels, signaling molecules, and the extracellular matrix (ECM) [7]. Cancer-associated fibroblasts (CAFs) are the most abundant stromal components in the TME, playing a pivotal role in this ecosystem and supporting cancer growth. Their roles in cancer development are diverse: regulation of ECM remodeling, immunosuppression, angiogenesis, metabolic reprogramming, metastasis and resistance to therapy [8]. Indeed, CAFs have been implicated in reducing the effectiveness of ER-targeted therapies and mediating resistance to these therapeutic modalities [9,10,11].

This manuscript is structured as a narrative review divided into sections covering the biology and heterogeneity of CAFs in breast cancer, CAF-mediated mechanisms of endocrine resistance, and potential therapeutic strategies targeting CAF-related pathways. Although this is a narrative review, a literature search was conducted using PubMed, Scopus, and Google Scholar databases to identify relevant studies published in English. Search terms included combinations of ‘cancer-associated fibroblasts’, CAFs’, estrogen receptor-positive breast cancer’, luminal breast cancer’, tumor microenvironment’, and ‘endocrine resistance’. Priority was given to experimental and mechanistic studies specifically investigating the role of CAFs in HR+ breast cancer.

## 2. CAF Heterogeneity in Breast Cancer

CAFs are key, heterogeneous components of the breast cancer TME, constituting up to 80% of the tumor mass [12]. Their role in breast cancer has been described for years; in 2011, Soon et al. described the role of CAFs in inducing the process of epithelial-to-mesenchymal transition (EMT) in breast cancer cells [13]. CAFs are a pivotal source not only of ECM components, but also of cytokines, chemokines, extracellular vesicles, and metabolites that are excreted in the TME and influence cancer development [14]. A reciprocal crosstalk between cancer cells and CAFs affects breast tumor progression. Paracrine transforming growth factor (TGF)-β1 signaling initiated from CAFs promotes EMT, enhancing the migratory capacity of breast cancer cells [15]. Similarly, CAFs form cell clusters with circulating tumor cells, helping them endure blood shear forces and facilitate metastatic colonization [16]. In terms of metabolism, the term “reverse Warburg effect” has been proposed to explain the role of CAFs in metabolically reprogramming the breast TME. Cancer cells create oxidative stress in CAFs, which in turn experience a metabolic shift to glycolysis, providing energy to support the oxidative phosphorylation of neighboring cancer cells [17,18]. Nevertheless, the role of CAFs in tumorigenesis and cancer progression has recently gained more attention in the scientific community.

CAFs in breast cancer originate from diverse sources; resident tissue fibroblasts, mesenchymal cells derived from bone marrow, adipocytes, endothelial cells, mesothelial cells covering body cavities, and pericytes are listed amongst the possible sources [14]. The foundational work by Costa et al. [19] identified four CAF subsets (CAF-S1 through CAF-S4) in human breast cancer using six concomitant markers, fibroblast activation protein (FAP), alpha-smooth muscle actin (α-SMA), cluster of differentiation (CD)29, fibroblast-specific protein 1 (FSP1), platelet-derived growth factor receptor (PDGFR)-b and caveolin-1 (CAV1). CAF-S2 and CAF-S3 resemble quiescent, normal-like fibroblasts, while CAF-S1 and CAF-S4 are tumor-restricted activated populations with distinct functions. CAF-S1 promotes immunosuppression, attracting and retaining regulatory T cells (Tregs) via CXC motif chemokine 12 (CXCL12), OX40 ligand (OX40L), programmed death-ligand 2 (PD-L2), and junctional adhesion molecule 2 (JAM2), while CAF-S4 promotes metastasis through contractile and migratory programs.

Single-cell transcriptomics further explored the heterogeneity of breast cancer CAFs, revealing additional functional phenotypes [14]. Myofibroblastic CAFs (myCAFs), characterized by high α-SMA and FAP expression, induce ECM remodeling, cause fibrotic stiffening of the surrounding tissue, hinder drug delivery by creating physical obstacles, and facilitate evasion of the immune system. In contrast, inflammatory CAFs (iCAFs) are responsible for shaping an immunosuppressive TME by secreting inflammatory factors, such as interleukin (IL)-6 and CXCL12, and by attracting immune cells that suppress the body’s defense response [14]. A subset of myCAFs, known as senescent CAFs (senCAFs), further supports immunosuppression, impairing natural killer (NK) cell cytotoxicity in the breast cancer TME [20]. Partners in this process, antigen-presenting CAFs (apCAFs), express major histocompatibility complex (MHC) class II molecules on their cell surface, possibly enabling antigen presentation. Other breast CAF subpopulations that have been described include vascular CAFs (vCAFs), associated with angiogenesis, circulating CAFs (cCAFs), linked to advanced disease stages, dividing CAFs (dCAFs), linked to cell cycle processes, and cluster of differentiation 63+ (CD63+) CAFs, associated with tamoxifen resistance, further emphasizing their functional heterogeneity [14].

CAFs also differ between breast cancer subtypes; for example, iCAFs are plentiful in patients with triple-negative breast cancer (TNBC), while myCAFs are enriched in patients with luminal breast cancer subtypes [14]. In luminal breast cancer, several CAF-related proteins, such as FSP1, podoplanin, prolyl 4-hydroxylase subunit alpha 3 (P4HA3), neuron-glial antigen 2 (NG2), and PDGFR-a have a lower expression rate [21].

CAF subtypes exist in a dynamic ecosystem, characterized by distinct spatial distribution and phenotypic switching governed by competing signaling axes from the TME. In the breast cancer TME, driven by spatial organization, several FAP-expressing myCAF clusters are observed close to cancer cells, while the FAP-expressing detoxification-associated iCAF (Detox-iCAF) cluster is located around blood vessels. An example of phenotypic plasticity is how breast cancer cells can adapt and change their behavior in response to their environment. The activation of dipeptidyl peptidase-4 (DPP4)- and (Yes-associated protein) YAP-dependent mechanisms mediates the transition of Detox-iCAF towards an immunosuppressive ECM-producing myCAF (ECM-myCAF) subtype [22]. A particularly important clinical consideration is that therapeutic interventions themselves can reshape CAF composition. In breast cancer patients receiving anti-programmed death (PD)-1 immunotherapy, responders show stromal remodeling characterized by functional re-education of iCAFs (transitioning to a pro-inflammatory CXCL9-CXCR3 axis) and concurrent disarmament of vCAF and myCAF populations. On the contrary, non-responders show stromal fortification of vCAFs and iCAFs, which provide supportive signaling to tumor cells and form a protective niche [23]. This demonstrates that CAF plasticity is not merely a biological curiosity but a determinant of treatment response.

## 3. The Role of CAFs in ER+ Breast Cancer

The role of CAFs in the TME of ER+ breast cancer is primarily characterized by their tumor-promoting roles. Even in patients with favorable prognoses and high survival rates, CAFs have been linked to metastatic facilitation. A specific CAF subtype, CAF-S1, characterized by the simultaneous expression of FAP and αSMA proteins, was found to be significantly enriched in early luminal breast cancer (T1N0), which subsequently developed distant metastases. The CAF-S1 pro-metastatic activity toward bone, the predominant metastatic site in luminal breast cancer, was mediated by the cadherin-11 (CDH11)/osteoblast cadherin axis [24].

With regards to CAF markers, elevated expression of platelet-derived growth factor receptor beta (PDGFRb) is linked to resistance to tamoxifen, as well as worse prognosis and tumor recurrence in breast and prostate cancer [25]. Nevertheless, specific CAF subtypes have been implicated in conferring endocrine resistance in breast tumors, which constitutes the focus of this review [9]. Defined by cluster of differentiation 146 (CD146) expression, two CAF subtypes have been identified in ER+ breast cancer [9]. CD146 is an adhesion molecule, primarily expressed in cells comprising vessels, that has been involved in endothelial dysfunction [26]. Brechbuhl et al. [9] demonstrated that CAFs lacking CD146 expression in ER+ breast cancer diminish tumor sensitivity to estrogen, thereby promoting resistance to tamoxifen treatment. On the contrary, CAFs expressing CD146 were found to maintain estrogen-dependent proliferation and support the sensitivity of cancer cells to tamoxifen.

CAFs have also been linked to the prognosis of breast cancer patients. In luminal breast cancer, intratumoral CAFs exhibiting high a-SMA expression are linked to poor survival outcomes [27]. Conversely, CAFs expressing cluster of differentiation 34 (CD34) are associated with improved prognosis when in abundance in the luminal A TME [28].

However, it is important to note that CAFs represent a double-edged component within the TME [29]. While many CAF populations are predominantly recognized for their pro-tumorigenic roles, distinct CAF subtypes exert favorable, tumor-suppressive effects, most notably by maintaining ER expression and tamoxifen sensitivity, suppressing tumorigenesis, and enhancing chemosensitivity [30]. The most well-characterized favorable CAF subtype in ER+ breast cancer is the CD146-expressing CAF [9].

## 4. CAF-Associated Mechanisms of Endocrine Resistance in HR+ Breast Cancer

Endocrine therapy resistance is a major challenge in breast cancer management. Driven by a diverse and often convergent set of mechanisms, ranging from ER pathway alterations, bypass signaling activation, cell cycle dysregulation, epigenetic reprogramming, metabolic adaptation, and TME remodeling [31], endocrine therapy resistance affects approximately 25% of those receiving adjuvant endocrine therapy [32].

Mutations in the ER gene *ESR1* are the most clinically significant acquired resistance mechanism. On the other hand, activation of alternative growth factor signaling pathways enables estrogen-independent proliferation. Phosphatidylinositol-3-kinase (PI3K)/protein kinase B (AKT)/mammalian target of rapamycin (mTOR) or mitogen-activated protein kinase (MAPK)/rat sarcoma (RAS)/rapidly accelerated fibrosarcoma (RAF) pathway hyperactivation are listed amongst the most common resistance mechanisms. Fibroblast growth factor receptor (FGFR) and *HER2* amplification or mutation have been also implicated in developing resistance to either endocrine therapy or CDK4/6 inhibition. Epigenetic reprogramming of ER transcription, upregulation of forkhead box A1 (FOXA1), cyclin D, c-myc, and altered expression of receptor tyrosine kinases (RTKs) can weaken the effects of antiestrogen treatments and stimulate pathways associated with proliferation and metastasis [31]. Lastly, YAP/transcriptional coactivator with PDZ-binding motif (TAZ) activation acts as a key driver of endocrine resistance in breast cancer by enabling cells to bypass ER signaling, leading to tumor survival and relapse [33].

### 4.1. Signaling Pathways Involved in CAF-Driven Endocrine Resistance

As mentioned earlier, development of anti-endocrine resistance in HR+ breast cancer has been associated with activation of signaling pathways that rely on the PI3K/AKT/mTOR pathway and involve molecules, such as epidermal growth factor receptor (EGFR), HER2, and insulin-like growth factor 1 receptor (IGF1R) [34,35,36]. Nevertheless, the exact mechanisms by which CAFs confer endocrine therapy resistance are not fully understood. Several mechanisms have been proposed, with the field rapidly evolving in the past few years.

A specific breast CAF subtype has been described in the literature, which expresses CD63 transmembrane protein, a key factor in extracellular vesicle production that may also serve as a potential prognostic marker in solid malignancies [37]. CD63-expressing CAFs secrete exosomal miRNA (miR)-22, which represses *estrogen receptor-a (ERa)* and *phosphatase and tensin homolog (PTEN)* genes in tumor cells, contributing to tamoxifen resistance [38]. Loss of *PTEN* leads to PI3K pathway activation, a pathway that has already been mentioned to be involved in endocrine resistance development [39]. In vitro experiments have validated this observation; coculture of human mammary progenitor CD63-expressing CAFs with MCF-7 breast cancer cells expressing ER has been shown to augment tamoxifen resistance [38]. Intriguingly, in vitro experiments displayed that when administering an anti-CD63 neutralizing monoclonal antibody (mAb) or crGd-Mir-22-sponge nanoparticles, sensitivity to tamoxifen was increased [38]. Also, it should be noted that CD63-expressing CAFs confer resistance to CDK4/6 inhibitors as well, further deteriorating the prognosis of patients with HR+ breast cancer [38].

Notably, CAFs have been found to reprogram the ER response in luminal breast cancer, facilitating estrogen independence [40]. Specifically, CAFs downregulate estrogen-dependent regulation of traditional ERα target genes, including *Progesterone Receptor (PGR), CXCL12* and *Myb proto-oncogene like 1 (MYBL1)*. On the contrary, they upregulate genes responsible for migration, invasion, metastasis and drug resistance. At first glance, it may seem contradictory that CAFs support ERα-driven breast tumors while simultaneously reducing the expression of ERα itself. Reid et al. [40] suggest that although CAFs reduce ERα levels, certain pro-tumorigenic downstream targets of this receptor remain active or are even enhanced by CAF signaling. It is still unclear whether CAF-mediated paracrine signaling maintains the expression of these genes through the same promoter elements used by ERα or through CAF-specific regulatory mechanisms. A functional drug screen identified signaling pathways, including TGF-β and JAK, as actionable targets to oppose CAF-induced modulation of ERα activity, offering new insights into combating the development of endocrine resistance in luminal breast cancer [40].

Compared to the nuclear ERα, which acts as a transcription factor (TF), the G protein-coupled estrogen receptor (GPER) is a membrane-bound receptor initiating rapid, non-genomic signaling [30]. GPER is a well-recognized mediator of tamoxifen resistance; while tamoxifen leads to ERα downregulation, GPER is upregulated, leading to increased estrogen production signaling [41]. Previous studies showed that GPER augments β1-integrin expression via the EGFR/extracellular regulated protein kinase (ERK) pathway, activating downstream kinases. In the presence of CAFs, these signaling pathways are more easily activated, and EMT is more easily provoked, ultimately leading to tamoxifen resistance [42].

Serving as important mechanosensors of the cell microenvironment and aberrantly expressed in the stroma [43], YAP and TAZ translocate to the CAF nucleus, binding to transcriptional enhancer-associated domain (TEAD) TFs, and activating oncogenic pathways. YAP and TAZ have been linked to stromal reprogramming, triggering endocrine resistance-associated mutations in the breast TME [44]. *ESR1* Y537S-mutant breast cancer cells reprogram normal fibroblasts into CAFs through a YAP1-dependent mechanism driven by paracrine insulin-like growth factor 1 (IGF-1) signaling [44]. Ultimately, CAF abundance leads to increased metastatic capacity of breast cancer cells, validating the existence of a tumor–stroma bidirectional crosstalk [44].

### 4.2. Epigenetic Mechanisms Involved in CAF-Driven Endocrine Resistance

Epigenetic mechanisms have been found to be implicated in bestowing resistance to endocrine therapy. miR-20 and miR-22 are well-described exosomal molecules involved in tamoxifen and abemaciclib resistance in HR+ breast cancer, respectively [38,45]. CAF-derived exosomes in the form of long non-coding RNAs (lncRNAs) have also been found to be responsible for developing tamoxifen resistance. RNA-sequencing techniques identified the lncRNA PRKCQ-AS1 to be upregulated in tamoxifen-resistant samples, which was confirmed not only in vitro, but also in vivo. Conversion of normal fibroblasts (NFs) to CAFs under the influence of TGF-β leads to increased paired Box 5 (PAX5) and PRKCQ-AS1 production in CAFs. The TF PAX5 augments PRKCQ-AS1 production, and PRKCQ-AS1, in turn, is transferred through exosomes in the cytoplasm of breast cancer cells, functioning as a molecular sponge for miR-200a-3p. Ultimately, increased expression of mitogen-activated protein kinase phosphatase 1 (MKP1) is provoked. Because tamoxifen typically triggers apoptosis in breast cancer cells, MKP1 interferes with this process by decreasing phosphorylation within the mitogen-activated protein kinase (MAPK)/c-Jun N-terminal kinase (JNK) signaling pathway. As a result, apoptosis of breast cancer cells is hampered, contributing to the development of tamoxifen resistance [46].

### 4.3. Paracrine Signaling Involved in CAF-Driven Endocrine Resistance

Other stroma-mediated resistance mechanisms include CAF-secreted cytokines, encompassing growth-regulated oncogene α (GROα) and chemokine (C-C motif) ligand 19 (CCL19) [47]. Notably, GROa secretion activates signal transducer and activator of transcription 3 (STAT3) and ERK pathways, which have already been described to be activated in endocrine-resistant breast cancer cells [48,49]. Patient-derived organoids (PDOs) and matching CAFs, from primary and metastatic HR+ breast cancers, have validated this observation [47]. In this context, IL-6 is one of the most extensively studied CAF-secreted cytokines. By activating the JAK/STAT3 pathway in breast cancer tumor cells, it promotes fulvestrant resistance [50]. The IL-6/STAT3 pathway triggers the production of miR-221 microvesicles, which are in turn transferred to tumor cells, downregulating ERα expression and enhancing de novo endocrine-resistant breast tumors [48]. CAFs also secrete multiple fibroblast growth factor (FGF) ligands, most prominently FGF2, FGF5, FGF7, and FGF17, that activate FGF receptor (FGFR) signaling on breast cancer cells through paracrine activity [51]. Multiple reports displayed a role of FGFR signaling in breast cancer biology and antiestrogen resistance [52,53]. Overexpression, gene copy number alterations, mutations and fusions in FGFRs promote antiestrogen resistance in breast cancer [53]. Similarly, FGF2 mediates resistance to estrogen deprivation therapy, mTOR and PI3K pathway inhibition, by upregulating ERK1/2 in ER-positive breast cancer [54].

### 4.4. ATP-Binding Cassette (ABC) Transporters and CAF-Driven Endocrine Resistance

ATP-binding cassette (ABC) transporters are membrane proteins that pump drugs out of cells, significantly contributing to anticancer therapy resistance [55]. Tamoxifen and other anti-estrogen agents have a well-documented connection with ABC transporters. Prolonged tamoxifen exposure upregulates ATP-binding cassette subfamily G member 2 (ABCG2) both at the mRNA and protein levels in MCF-7 breast cancer cells, which was also confirmed in tamoxifen-resistant patient tumor tissues [56]. The same ABC transporter was found overexpressed in 200 patients who received neoadjuvant endocrine therapy, predicting the development of resistance to subsequent chemotherapy treatments [57]. The correlation between endocrine resistance and ABC transporters has already been established. However, it is important to emphasize that ABC transporters may also represent a significant yet underappreciated mechanistic link in CAF-driven endocrine resistance.

Several CAF-secreted factors have been identified as upstream regulators of ABC transporters. CAFs are a major source of IL-6 in the TME [50]. IL-6, in turn, leads to increased ABCG2 protein expression [58,59]. As noted earlier, CAFs secrete miR-22, which leads to loss of *PTEN* and subsequently, the evolution of tamoxifen resistance. Notably, *PTEN* inactivation leads to increased ABCG2 expression through activation of the PI3K/AKT pathway, which may contribute to the development of tamoxifen resistance [60]. Another proposed mechanism is that the overexpression of miR-22 by CAFs mediates the methylation of ABC transporter promoters, eventually, and with non-well-defined mechanisms leading to their overactivation [61]. Mechanistically, β1-integrin binding to collagen type 1, which is a major ECM component deposited by CAFs, activates several ABC transporters, such as ABCG2, P-glycoprotein (P-gp), and multidrug resistance-associated protein 1 (MRP1) in breast cancer cells [62]. The evidence collectively demonstrates that CAF-secreted factors, CAF-generated TME conditions, and CAF-activated signaling pathways all converge on ABC transporter upregulation. The exact mechanisms underlying this observation are not fully elucidated; rather, they are based on theoretical insights. Addressing this gap could offer clinical implications for treatment sequencing, particularly regarding the choice of chemotherapy agents after endocrine resistance arises.

Table 1 presents a summary of the mechanisms involved in CAF-mediated endocrine resistance in HR+ breast cancer.

## 5. Therapeutic Strategies Involving CAFs to Enhance Endocrine Therapy Outcomes

Given the pertinent role of CAFs in mediating endocrine therapy resistance, CAFs are now considered attractive targets for anticancer therapeutic strategies. However, several challenges are currently present, spanning from the identification of CAF-specific markers to their functional heterogeneity and plasticity. Despite these hurdles, an increasing number of preclinical studies have focused on CAF targeting to improve anti-cancer treatment, while some clinical trials involving CAF targeting agents are already ongoing. Aiming to enhance hormonal blockage effectiveness in breast cancer, the proposed strategies include CAF depletion, interfering with CAF-secreted cytokines, and CAF reprogramming towards an antitumorigenic phenotype.

FAP represents the most widely exploited CAF marker, expressed on CAFs in the majority of solid tumors [63]. Although FAP is expressed also in smooth muscle and epithelial cells and is not considered solely a CAF-specific marker [64], multiple strategies have been developed to target CAFs by focusing on targeting FAP, such as antibody-drug conjugate (ADCs) and chimeric antigen receptor (CAR)-T cells [65]. OMTX705 is a novel FAP-targeting ADC that has shown a satisfying antitumor profile in TNBC patient-derived xenograft models as a monotherapy agent and in combination with other anticancer agents [66]. Nonetheless, with regard to HR+ breast cancer, evidence is restricted, which could be explained by the fact that FAP expression is lower in luminal A breast cancer compared to other subtypes [67]. In targeted radiotherapeutics, a novel actinium-225 FAP-directed radiopharmaceutical therapy was explored in nine metastatic breast cancer patients (three luminal A, three luminal B, two HER2+, one TNBC) with otherwise limited therapeutic options. Although the number of patients is limited, encouraging survival outcomes ranging up to two years were observed [68]. Other radionuclide options include 177Lu-FAP-2286 peptide. Baum et al. [69] administered two cycles of 177Lu-FAP-2286 at a cumulative dose of 5.8 GBq to four patients (three luminal subtypes, one unknown). Two patients exhibited progressive disease, while two patients demonstrated stable disease, respectively. Similar findings have been reported by other researchers; yet, those studies involved heavily pretreated patients [70], whereas in this advanced setting, endocrine therapy has no established role.

As mentioned earlier in this review, two distinct CAF subtypes in breast cancer have been identified based on CD146 expression. The same study showed that these fibroblast subtypes can dictate which RTKs are active in the HR+ breast TME. For instance, while both CD146-positive and CD146-negative CAFs enhance EGFR phosphorylation in ER-positive breast cancer cells, CD146-negative fibroblasts additionally induce IGF1R phosphorylation and increase the sensitivity of these cells to the HER2 inhibitor trastuzumab [9]. These data suggest that, based on the CAF subtype that is abundant on the TME, endocrine treatment could be combined with the appropriate RTK inhibitor; TMEs abundant in CD146-negative CAFs could benefit from a broad RTK inhibitor simultaneously targeting EGFR, HER2 and IGFR1, while TMEs abundant in CD146-positive CAFs could derive clinical benefit from a single EGFR RTK inhibitor combined with endocrine treatment [9]. Notably, considering the functional heterogeneity of CAFs, precisely targeting selective CAF subpopulations could yield antitumor results. CAFs lacking CD146 expression or maintaining CD63 expression have been linked to dismal antiestrogen therapy outcomes. Efforts should be oriented towards targeting these specific subpopulations, while preserving the ones that exhibit an antitumorigenic phenotype.

Integrating the identification of CAF biomarker techniques into standard histopathological examinations could dictate possible treatment combinations. Artificial intelligence (AI) is increasingly important in identifying such biomarkers. Machine learning recently distinguished FXYD Domain Containing Ion Transport Regulator 1 (FXYD1), Sulfatase 1 (SULF1), and Tenascin XB (TNXB) as refined biomarkers with differential expression between luminal and non-luminal breast cancer, offering novel insights into precision breast cancer therapy [71]. Additionally, since breast cancer cells frequently rely on the signaling of HER2 to drive their proliferation independently of estrogen signaling, HER2 inhibition is another appealing option in luminal A breast cancer management. Combining HER2 inhibitors with already established endocrine therapeutic modalities could enhance endocrine blockage effectiveness [39], even at earlier stages where HER2 upregulation has not occurred yet.

Targeting cellular metabolism is becoming a promising strategy to overcome drug resistance in cancer therapy [72]. Glutamine produced by CAFs triggers resistance to tamoxifen in breast tumor cells, facilitating their survival [73]. Several mechanisms have been proposed to target CAF metabolism, aiming to overcome drug resistance in breast cancer therapy [74]. Statin drugs have shown promise in reducing breast cancer cell invasion by blocking the formation of 27-hydroxycholesterol, which promotes estrogen-dependent breast cancer growth [75]. Simultaneously, statins interfere with the Hippo pathway, impairing the nuclear localization of YAP/TAZ [76]. Targeting the Hippo pathway alongside administering endocrine treatment could significantly overcome endocrine resistance development [77]. Several observational trials have validated the positive effects of statin administration alongside endocrine therapy [78]. Phase II clinical trials are ongoing (NCT02958852, NCT03192293), evaluating concurrent statin administration with endocrine therapy. Prospective randomized trial data are awaited.

FGFR pathway inhibition initially represented a promising indirect CAF-targeted strategy. FGFR inhibition in combination with endocrine therapy showed satisfying antitumor results in vitro [79] and subsequently transitioned to the clinical trial setting. The phase II RADICAL trial (NCT01791985) evaluated AZD4547, a potent and selective inhibitor of FGFR1-3, with anastrozole or letrozole in ER-positive metastatic breast cancer patients who developed resistance to AIs. The objective response rate (ORR) was as low as 10%, with five patients achieving a partial response (pR) [80]. Tasurgratinib (E7090), another FGFR1-3 inhibitor, combined with fulvestrant in a phase I clinical trial (NCT04572295) showed a manageable safety profile in ER-positive, HER2−breast cancer patients who had previously received a CDK4/6 inhibitor. With an ORR of 21.9%, tasurgratinib demonstrated promising preliminary antitumor activity, with three out of four responders in the dose-escalation cohort remaining on treatment for more than 2 years [81]. Designed under the same rationale, NCT03238196 is a phase I study of the pan-FGFR inhibitor erdafitinib in combination with fulvestrant and the CDK4/6 inhibitor palbociclib in HR+, HER2− metastatic breast cancer patients. The ORR was determined at 65%, with three pRs observed amongst them. One pR lasted more than 2.5 years [81].

Several factors complicate FGFR-targeted therapy in breast cancer. Biomarker analysis performed in the NCT03238196 trial showed that high FGFR1 expression by immunohistochemistry (IHC) correlated with longer progression-free survival (PFS) rates. However, FGFR1 amplification did not predict response to treatment with the pan-FGFR tyrosine kinase inhibitor erdafitinib [82]. FGFR1 gene amplification and protein overexpression are discordant in >20% of patients, and both independently predict poor prognosis. Gene amplification testing alone may be insufficient; protein expression and stromal context should be complementary assessed [51]. Biomarker-driven patient selection has been further investigated in vitro; FGFR-amplified PDOs respond to treatment with fulvestrant, palbociclib, and rogaratinib, another pan-FGFR inhibitor, only when *phosphatidylinositol-4,5-bisphosphate 3-kinase catalytic subunit alpha (PIK3CA)* and *estrogen receptor 1 (ESR1)* mutations are not detected. In a dose-escalation trial including nine eligible patients harboring FGFR alterations, the triplicate combination of fulvestrant, palbociclib, and rogaratinib showed antitumor activity specifically in *PIK3CA*- and *ESR1*-wild type patients (PFS 9.1 vs. 1.9 months; *p* = 0.0005). *PIK3CA* and *ESR1* mutations appear to disadvantage FGFR inhibition, suggesting these should be used as exclusion biomarkers [83]. Notably, National Comprehensive Cancer Network (NCCN) guidelines, acknowledging FGFR targeting as a tumor-agnostic approach, propose erdafitinib as category 2B in breast cancer patients harboring *FGFR1-3* fusions or alterations [3]. Lack of established patient selection criteria, in addition to the early use of non-selective inhibitors, are possible reasons explaining the failure of FGFR inhibitors in clinical practice to date. Biomarker-guided approaches are warranted, aiming to improve FGFR-targeted therapies and successfully implement them in clinical practice.

Other prospective strategies targeting the paracrine function of CAFs have been also proposed. At a preclinical level, IL-6 receptor (IL-6R) (IL-6R) knockdown inhibited IL-6/STAT3 signaling and reversed tamoxifen resistance in breast cancer, both in vitro and in vivo [84]. Similarly, targeting PDGF chemokines with the PDGFR inhibitors sunitinib and ponatinib and combining this dual inhibition with tamoxifen seems to effectively hamper estrogen-dependent breast cancer growth in vitro [85]. CAF-derived exosomal lncRNA PRKCQ-AS1 and MKP1 protein, both contributing to CAF-supported tamoxifen resistance, may serve as novel therapeutic targets for luminal breast cancer patients receiving tamoxifen treatment [46].

Table 2 presents a comprehensive summary of the proposed therapeutic strategies against CAFs in HR+ breast cancer.

**Figure 1:**
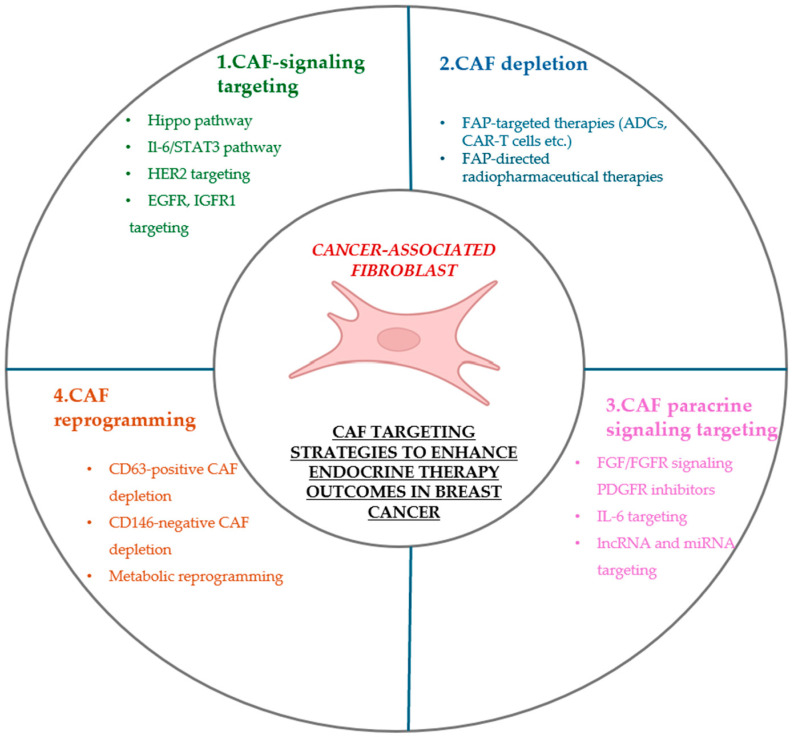
Potential therapeutic approaches involving CAFs aimed at improving endocrine therapy outcomes in HR+ breast cancer. 1. CAF signaling targeting (Hippo, IL-6/STAT3, HER2, EGFR, IGFR1 signaling pathways). 2. CAF depletion (FAP-targeting therapies, FAP-directed radiopharmaceutical therapies) 3. CAF paracrine targeting (FGF/FGFR signaling, PDGFR inhibitors, IL-6 inhibitors, lncRNA/miRNA targeting). 4. CAF reprogramming. (metabolic reprogramming of CAFs, selective depletion of CAF subpopulations-CAFs expressing CD63 or lacking CD146 have been implicated in conferring tamoxifen resistance). CAFs = cancer-associated fibroblasts, CAR-T cells = chimeric antigen receptor T cells, CD146 = cluster of differentiation 146, CD63 = cluster of differentiation 63, EGFR = epidermal growth factor receptor, FAP = fibroblast activation protein, FGF = fibroblast growth factor, FGFR = FGF receptor, HER2 = Human Epidermal Growth Factor Receptor 2, HR+ = hormone receptor positive, IGF1R = insulin-like growth factor 1 receptor, IL-6 = interleukin 6, lncRNA = long non-coding RNA, miRNA = microRNA, PDGFR = platelet-derived growth factor receptor, STAT3 = signal transducer and activator of transcription 3.

Figure 1 provides a schematic overview of potential therapeutic approaches involving CAFs aimed at improving endocrine therapy outcomes in HR+ breast cancer.

## 6. Conclusions

Endocrine resistance is a major obstacle in the management of HR+ breast cancer. Novel molecules blocking or modulating estrogen signaling are constantly being developed. Exploiting the TME has emerged as a promising strategy. The TME contributes as strongly to therapeutic resistance as malignant cells themselves, positioning CAFs as a central focus of breast cancer research. Indeed, CAFs have been involved in conferring endocrine resistance in luminal breast cancer. However, they represent a highly heterogeneous population, spanning from tumor-promoting to tumor-restraining phenotypes. Concentrating on eliminating the tumor-promoting subtypes and selectively preserving the tumor-restraining ones appeals to be an aspiring strategy. Although significant advancements have been made towards understanding the role of CAFs in breast cancer, a significant gap needs to be elucidated before their clinical application can be established.

## Figures and Tables

**Table 1:** Summary of CAF-associated mechanisms involved in endocrine resistance in HR+ breast cancer.

Mechanism	Core Concept	Description
Signaling pathway activation/ER signaling reprogramming	PI3K/AKT/mTOR, GPER/EGFR/ERK, Hippo pathways, ERα modulation	CAFs → miR-22 → PTEN loss → PI3K/AKT/mTOR activation CAFs → GPER upregulation → EGFR/ERK pathway activation → ↑ β1-integrin expression → downstream kinase activation Y537S-ERα → IGF-1 → IGF-1R → YAP1 → CAF abundance → metastasis CAFs → ERα target gene downregulation (PGR, CXCL12, MYBL1)
Epigenetic modifications	miR-22, lncRNA PRKCQ-AS1	CAFs → miR-22 → PTEN loss CAFs → TGFβ → ↑ PAX5 and PRKCQ-AS1 production → ↑ MKP1 → MAPK/c-JNK pathway activation → ↓ breast cancer cell apoptosis
Paracrine signaling	CAF-secreted cytokines (GROα, CCL19, IL-6), FGFR signaling	CAFs → GROα → JAK/STAT3 pathway CAFs → IL-6/STAT3 pathway → ↑ miR-221 → ERα downregulation (i) CAFs → FGFs → FGFR signaling activation → FGFR overexpression, mutation, fusion →antiestrogen resistance (ii) FGF2 → ERK1/2 upregulation → antiestrogen resistance
ABC transporters	ABCG2, P-gp, and MRP1 upregulation	CAFs → miR-22 → PTEN loss → ABCG2 upregulation CAFs → Collagen type I deposition (ECM) → β1-integrin activation in breast cancer cells → Activation of ABC transporters (ABCG2, P-gp, MRP1)

Abbreviations: ABC, ATP-binding cassette; ABCG2, ATP-binding cassette subfamily G member 2; AKT, protein kinase B; CAFs, cancer-associated fibroblasts; CCL19, chemokine C C motif ligand 19; c-JNK, c Jun N-terminal kinase; CXCL12, CXC motif chemokine 12; ECM, extracellular matrix; EGFR, epidermal growth factor receptor; ER, estrogen receptor; ERα, estrogen receptor α; ERK, extracellular regulated protein kinase; FGFR, fibroblast growth factor receptor; GPER, G protein-coupled estrogen receptor; GROα, growth-regulated oncogene α; IL-6, interleukin 6; JAK, Janus kinase; lncRNA, long non-coding RNA; MAPK, mitogen-activated protein kinase; miR-22, miRNA-22; miR-221, miRNA-221; MKP1, mitogen-activated protein kinase phosphatase 1; MRP1, multidrug resistance associated protein 1; mTOR, mammalian target of rapamycin; MYBL1, Myb proto oncogene like 1; PAX5, paired Box 5; P-gp, P-glycoprotein; PI3K, phosphatidylinositol 3 kinase; PGR, progesterone receptor gene; PTEN, phosphatase and tensin homolog; STAT3, Signal transducer and activator of transcription 3. Symbols: ↑ = Increase; ↓ = Decrease.

**Table 2:** Prospective therapeutic options against CAFs in HR+ breast cancer.

Therapeutic Strategy	Description	Stage (Preclinical/Clinical)	Limitations
FAP-targeting techniques	ADCs, CAR-T cells	Clinical	TNBC-oriented, FAP expression is lower in luminal A breast cancer compared to other subtypes
	FAP-targeted radionuclide options (^225^Ac-3BP-3940, 177Lu-FAP-2286 peptide)	Clinical	Current approaches include heavily pretreated patients, where endocrine treatments have no established role
CAF reprogramming	CD146-negative CAF–rich TMEs: endocrine therapy + broad RTK inhibition (targeting EGFR, HER2, and IGF1R) vs. CD146-positive CAF–rich TMEs: endocrine therapy combined + selective EGFR inhibition.	Preclinical	CAF heterogeneity, identification of CAF biomarker techniques into standard histopathological examinations, data quality, validation, and implementation challenges
	Targeting CD63-expressing or CD146-lacking CAFs		
CAF metabolism	Statin administration concurrent with antiestrogen treatment	Clinical	Lack of prospective randomized trial evidence, phase II clinical trials are ongoing (NCT02958852, NCT03192293)
FGFR signaling targeting	FGFR inhibitors	Clinical	Lack of established patient selection criteria, early use of non-selective inhibitors
Paracrine targeting	IL-6R inhibitors, PDGFR inhibitors, CAF-derived exosomes targeting	Preclinical	Preclinical and clinical validation are expected.

Abbreviations: CAFs = cancer-associated fibroblasts, CAR-T cells = Chimeric Antigen Receptor T cells, CD146 = cluster of differentiation 146, CD63 = cluster of differentiation 63, EGFR = epidermal growth factor receptor, FAP = fibroblast activation protein, FGFR = FGF receptor, HER2 = Human Epidermal Growth Factor Receptor 2, HR+ = hormone receptor positive, IGF1R = insulin-like growth factor 1 receptor, IL-6R = interleukin 6 receptor, PDGFR = platelet-derived growth factor receptor, TNBC = triple-negative breast cancer.

## Data Availability

No new data were created or analyzed in this study. Data sharing are not applicable to this article.

## Abbreviations

ABC	ATP-binding cassette
ADC	antibody drug conjugate
AIs	aromatase inhibitors
AKT	protein kinase B
apCAFs	antigen-presenting CAFs
CAFs	cancer-associated fibroblasts
CCL19	chemokine C C motif ligand 19
cCAFs	circulating CAFs
CD146	cluster of differentiation 146
CD4/6	Cyclin Dependent Kinases 4 and 6
CD63+	cluster of differentiation 63+
CDH11	cadherin-11
CXCL12	CXC motif chemokine 12
dCAFs	dividing CAFs
DPP4	dipeptidyl peptidase-4 ECM extracellular matrix
EGFR	epidermal growth factor receptor
EMT	epithelial to mesenchymal transition
ER	estrogen receptor
ERα	estrogen receptor α
ERK	extracellular regulated protein kinase
ESR1	estrogen receptor 1
FAP	fibroblast activation protein
FGF	fibroblast growth factor
FGFR	FGF receptor
FSP1	fibroblast-specific protein 1
FXYD1	distinguished FXYD Domain Containing Ion Transport Regulator 1
GPER	G protein-coupled estrogen receptor
GROα	growth-regulated oncogene α
HER2−	human epidermal growth factor receptor 2 negative
HR+	hormone receptor positive
IGF1R	insulin-like growth factor 1 receptor
IHC	immunohistochemistry
IL	interleukin
iCAFs	inflammatory CAFs
JAM2	junctional adhesion molecule 2
JNK c	Jun N-terminal kinase
lncRNAs	long non-coding RNAs
MAPK	mitogen-activated protein kinase
mAb	monoclonal antibody
MHC	major histocompatibility complex
miR	miRNA
MKP1	mitogen-activated protein kinase phosphatase 1
mTOR	mammalian target of rapamycin
MYBL1	Myb proto oncogene like 1
myCAFs	myofibroblastic CAFs
NFs	normal fibroblasts
NK	natural killer
NG2	neuron glial antigen 2
ORR	objective response rate
OX40L	OX40 ligand
P4HA3	prolyl 4 hydroxylase subunit alpha 3
PAX5	paired Box 5
PDGFRa	platelet-derived growth factor receptor alpha
PDGFRb	platelet-derived growth factor receptor beta
PD-1	anti-programmed death
PD-L2	programmed death-ligand 2
PDOs	patient-derived organoids
PFS	progression-free survival
PI3K	phosphatidylinositol 3 kinase
PIK3CA	phosphatidylinositol 4,5 bisphosphate 3 kinase catalytic subunit alpha
PR	progesterone receptor
pR	partial response
PTEN	phosphatase and tensin homolog
RTKs	receptor tyrosine kinases
SERDs	selective estrogen receptor degraders
SERMs	selective estrogen receptor modulators
STAT3	signal transducer and activator of transcription 3
SULF1	sulfatase 1
TAZ	transcriptional coactivator with PDZ-binding motif
TEAD	transcriptional enhanced associate domain
TF	transcription factor
TGF	transforming growth factor
TME	tumor microenvironment
TNBC	triple negative breast cancer
TNXB	Tenascin XB
vCAFs	vascular CAFs
α SMA	alpha smooth muscle actin
